# Evidence of a Cellulosic Layer in *Pandoravirus massiliensis* Tegument and the Mystery of the Genetic Support of Its Biosynthesis

**DOI:** 10.3389/fmicb.2019.02932

**Published:** 2019-12-20

**Authors:** Djamal Brahim Belhaouari, Jean-Pierre Baudoin, Franck Gnankou, Fabrizio Di Pinto, Philippe Colson, Sarah Aherfi, Bernard La Scola

**Affiliations:** ^1^Microbes, Evolution, Phylogeny and Infection (MEPHI), UM63, Institut de Recherche pour le Développement (IRD), Assistance Publique - Hôpitaux de Marseille (AP-HM), Aix-Marseille University, Marseille, France; ^2^IHU Méditerranée Infection, Marseille, France

**Keywords:** *Pandoravirus*, giant virus, capsid, cellulose, cellulose synthase

## Abstract

Pandoraviruses are giant viruses of ameba with 1 μm-long virions. They have an ovoid morphology and are surrounded by a tegument-like structure lacking any capsid protein nor any gene encoding a capsid protein. In this work, we studied the ultrastructure of the tegument surrounding *Pandoravirus massiliensis* virions and noticed that this tegument is composed of a peripheral sugar layer, an electron-dense membrane, and a thick electron-dense layer consisting in several tubules arranged in a helicoidal structure resembling that of cellulose. *Pandoravirus massiliensis* particles were stained by Calcofluor white, a fluorescent dye of cellulose, and the enzymatic treatment of particles by cellulase showed the degradation of the viral tegument. We first hypothesized that the cellulose tegument could be synthesized by enzymes encoded by the virus. Bioinformatic analyses revealed in *P. massiliensis*, a candidate gene encoding a putative cellulose synthase, with a homology with the BcsA domain, one of the catalytic subunits of the bacterial cellulose synthase, but with a low level of homology. This gene was transcribed during the replicative cycle of *P. massiliensis*, but several arguments run counter to this hypothesis. Indeed, even if this gene is present in other pandoraviruses, the one of the strain studied is the only one to have this BcsA domain and no other enzymes involved in the synthesis of cellulose could be detected, although we cannot rule out that such genes could have been undetected among the large proportion of Orfans of pandoraviruses. As an alternative, we investigated whether *P. massiliensis* could divert the cellulose synthesis machinery of the ameba to its own account. Indeed, contrary to what is observed in the case of infections with other giant viruses such as mimiviruses, it appears that the transcription of the ameba, at least for the cellulose synthase gene, continues throughout the growth phase of particles of *P. massiliensis*. Finally, we believe that this scenario is more plausible. If confirmed, it could be a unique mechanism in the virosphere.

## Introduction

Giant viruses of ameba are phylogenetically closely relative to the Nucleo – Cytoplasmic Large DNA Viruses (NCLDVs), within a new proposed order named Megavirales (Colson et al., 2013). Since the discovery of the first giant virus of ameba in La Scola et al. (2003), dozens of new giant viruses were described, considerably expanding our knowledge about their diversity, structure, genomics and evolution.

In 2013, two new complex giant viruses, named pandoraviruses, were described (Philippe et al., 2013). They replicate in *Acanthamoeba castellanii* and compose a new phylogenetic group of giant viruses of ameba related to phycodnaviruses. The first isolate, named *Pandoravirus salinus*, originated from a marine sediment layer of the Tunquen River in Chile. The second isolate, *Pandoravirus dulcis*, was isolated from the mud of a freshwater pond in Australia. Pandoraviruses harbor specific morphological and genetic features, including ovoid-shaped particles with an ostiole-like apex and measuring ∼1.0 μm in length and ∼0.5 μm in diameter, classifying them as the second largest particles after those of Pithovirus sibericum (Legendre et al., 2014). These viruses have a double-stranded DNA genome, up to 2.5 Mb (for *P. salinus*), the largest genome ever described to date in the virosphere (Philippe et al., 2013). Subsequently, the nature of an endocytobiont of *Acanthamoeba* isolated few years before in Germany from the contact lens and storage case fluid of a patient with keratitis, was recognized as the third *Pandoravirus* and was named *Pandoravirus inopinatum* (Scheid, 2016; Scheid et al., 2014). In 2015–2016, we isolated three new *Pandoravirus* strains from sewage and soda lake water samples collected in Brazil, by co-culture on *A. castellanii*. These viruses were named, respectively, *P. massiliensis*, *Pandoravirus pampulha*, and *Pandoravirus braziliensis* (Aherfi et al., 2018). Other recent prospecting studies reported the isolation of seven additional strains: *Pandoravirus quercus*, isolated from a soil sample collected in Marseille (France); *Pandoravirus neocaledonia*, isolated from the brackish water of a mangrove near Noumea airport (New Caledonia), *Pandoravirus macleodensis*, isolated from a freshwater pond near Melbourne (Australia) (Legendre et al., 2018), *Pandoravirus celtis*, isolated from a soil sample collected in Marseille (Legendre et al., 2019) and three new pandoraviruses isolated from water samples in Brazil (Pereira Andrade et al., 2019).

Although other giant viruses with a similar ovoid morphology have been described, including pithoviruses, cedratviruses or Orpheovirus (Legendre et al., 2014; Andreani et al., 2016, 2018), pandoraviruses have the intriguing particularity to harbor no hints of a known capsid protein and, at the same time, the absence in virions of any structure similar to that of a known capsid (Philippe et al., 2013; Scheid, 2016; Aherfi et al., 2018; Legendre et al., 2018). In addition, no capsid-resembling protein was identified by proteomics in the *P. salinus* and *P. massiliensis* virions (Philippe et al., 2013; Aherfi et al., 2018). *Pandoravirus* virions are wrapped in a ≈70 nm-thick tegument-like envelope, composed of three layers: one ≈20 nm-thick of light density; one intermediate of ≈25 nm-thick that appears darker and composed of fibrils; and an external layer ≈25 nm-thick with a medium density (Philippe et al., 2013). At one of the particle apex, an aperture of ≈70 nm in diameter opens the viral tegument. An internal lipid membrane is present beneath the tegument, thus delimiting the particle core. The tegument and the internal content of *Pandoravirus* virions are synthesized simultaneously, from the aperture-harboring apex, during neo-virion synthesis in the cytoplasm of the ameba. However, the nature of this tegument has remained unknown to date. Its deciphering is needed to refine the definition of these giant viruses.

In the present study, we aimed to characterize the nature of the tegument of pandoraviruses.

## Materials and Methods

### Transmission Electron Microscopy, Electron Tomography and Scanning Electron Microscopy

For negative staining, a drop of purified *P. massiliensis* particles fixed with 2.5% glutaraldehyde in 0.1 M cacodylate buffer was adsorbed onto formvar carbon films on 400 mesh nickel grids (FCF400-Ni, EMS). Grids were stained for 10 seconds with 1% molybdate solution in filtered water at room temperature. For sections, *Pandoravirus* particles were ultracentrifugated at 8,000 × *g* for 10 min and fixed for 1 h at 4°C under gentle mixing after pellet resuspension in a mixture of 1.2% glutaraldehyde/0.05% ruthenium red in 0.1 M cacodylate buffer. Virus particles were ultracentrifugated at 8,000 × *g* for 10 min to discard supernatant and were then fixed for 3 h at 4°C in a mixture of 1.2% glutaraldehyde/0.05% ruthenium red in 0.1 M cacodylate buffer. Then, *P. massiliensis* virions were washed thrice 10 min with 0.1 M Cacodylate buffer at 4°C. Next steps were performed at room temperature. Viral particles were rinsed twice for 15 min each, with a cacodylate 0.1 M/saccharose 0.2 M in water solution, and were dehydrated with ethanol 50, 70, and 96%, for 15, 30, and 30 min, respectively. *Pandoravirus massiliensis* virions were then placed for 1 h in a mix of LR-White resin 100% (Ref. 17411, MUNC-500; Polysciences) and ethanol 96% in a 2:1 ratio. After 30 min in pure 100% LR-White resin, particles were placed in 100% LR-White resin overnight at room temperature. *Pandoravirus* particles were then placed for 1 h in 100% resin at room temperature. A total of 1.5 mL of Pure 100% LR-White resin was added on the virus pellet. Polymerization was achieved at 60°C for 3 days. Between all steps of inclusion, the samples were ultracentrifuged at 2,400 × *g* and the supernatant was discarded. Sections of 70 or 300 nm-thick were cut on a UC7 ultramicrotome (Leica). Ultrathin sections were deposited on 300 mesh copper/rhodium grids (Maxtaform HR25, TAAB). They were post-stained with 5% uranyl acetate and lead citrate according to the Reynolds method (Reynolds, 1963). Gold nanoparticles with a diameter of 10 nm (Ref.752584; Sigma-Aldrich) were deposited on both faces on the 300 nm thick ultrathin sections for tomographic fiducial alignment. Electron micrographs were acquired on a Tecnai G^2^ transmission electron microscope operated at 200 keV and equipped with a 4096 × 4096 pixel resolution Eagle camera (FEI). Tomography tilt series were acquired with the Explore 3D (FEI) software for tilt ranges of 110 with 1 increments. The mean applied defocus was – 4 μm. The magnification ranged between 9,600 and 29,000 with pixel sizes between 1.12 and 0.37 nm, respectively. The average thickness of the tomograms obtained was 155 ± 43 nm (*n* = 8 measures). The tilt-series were aligned using ETomo from the IMOD software package (University of Colorado, United States) (Kremer et al., 1996) by cross-correlation. The tomograms were reconstructed using the weighted-back projection algorithm in ETomo from IMOD (Kremer et al., 1996). ImageJ software was used for image processing (Schneider et al., 2012). For scanning electron microscopy, virions particles were deposited on glass slides via cytospin centrifugation and observed in a tabletop TM4000Plus (Hitachi, Japan) scanning electron microscope after air-drying.

### Cellulose Staining and Light Microscopy

Calcofluor staining was achieved by depositing a drop of purified *Pandoravirus* particles in PAS medium onto a glass slide and immediately adding 50 μL of calcofluor white (Ref.18909; Sigma-Aldrich) and 50 μL of 10% KOH prior to glass slide covering and confocal imaging. *Pandoravirus* particles were imaged with a Plan-Aprochromat ×63/1.4 immersion objective on a AiryScan LSM800 confocal laser scanning microscope (Zeiss). Image size was 512 × 512 pixels and scan zoom ranged from ×0.5 to ×2.9. Laser excitation with a 405 nm wavelength was used for calcofluor staining imaging and was coupled to an ESID detector for depicting particles contours.

### Enzymatic Treatment of *Pandoravirus massiliensis* Virions

A total of 50 μL of purified virions was added to 1 mL of cellulase solution (cellulase from *Trichoderma reesei*, aqueos, Sigma Aldrich, C2730-50ML) at different concentrations and incubated for 48 h at 45°C. In a second time, these samples were imaged on AiryScan LSM800 microscope confocal laser scanning microscope. The effects were then observed by confocal microscopy (AiryScan LSM800), scanning microscopy and transmission electronic microscopy. The number of intact viral particles was estimated using the imageJ software (Schneider et al., 2012). The infectivity of the *Pandoravirus* was assessed before and after the cellulase treatment by calculating the TCID50 using the method of Reed and Muench (1938). The DNA of particles was labeled by using Hoechst 33342 (NC: 62249, Thermofisher Solution) after the cellulase treatment to visualize the effect on DNA.

### Bioinformatic Analyses to Search for Cellulose Synthase Candidate Genes in the *Pandoravirus massiliensis* Genome

Sequences of the predicted ORFs of *P. massiliensis* in amino acids were used for BLAST searches against the NCBI GenBank nr database. The analyses were performed using 1e-2, 25 and 50% as thresholds for the evalue, the homology and the coverage of aligned sequences, respectively. Phylogenetic reconstruction was performed using the Maximum Likelihood method with the MEGA6 software (Tamura et al., 2013). Conserved domains were also searched for by DELTA-BLAST analyses against the Conserved Domain Database (CDD) (Marchler-Bauer et al., 2017).

BLASTp, tBLASTn and BLASTn analyses were also performed against the gene contents and complete genomes of the 9 other pandoraviruses, i.e., *Pandoravirus dulcis*, *P. salinus* (Philippe et al., 2013), *P. inopinatum* (Scheid et al., 2014; Scheid, 2016), *P. quercus* (Legendre et al., 2018), *P. macleodensis* (Legendre et al., 2018), *P. neocaledonia* (Legendre et al., 2018), *P. celtis* (Legendre et al., 2019), *P. braziliensis* and *P. pampulha* (Aherfi et al., 2018).

### Transcription of Candidate Gene of Cellulose Synthase of *Pandoravirus massiliensis* and Cellulose Synthase of *Acanthamoeba castellani*i

RNA was extracted with the RNeasy mini kit (Cat No: 74104, Qiagen, France) at different time points of the *P. massiliensis* cycle, from H0 (i.e., 45 min after the infection of ameba cells by viral particles) until H12 post-infection (release of neo-synthetized virions). Total RNA was eluted in 50 μL of RNase-free water; 2.5 μl of RNaseOUT (Thermo Fisher Scientific, France) was added to the eluate to discard RNase. The DNase treatment was performed with TURBO DNase (Invitrogen, France; six cycles of 30 min incubation at 35°C). Two PCR systems targeting the DNA polymerase gene of *P. massiliensis* (forward primer: 5′-ATGGCGCCCGTCTGGAAG; reverse primer: 5′-GGCGCCAAAGTGGTGCGA) and the housekeeping gene of the RNA polymerase of *A castellanii* (forward: 5′-ACGAACTTCCGAGAGATGCA; reverse: 5′-CACCTTGACCAGTCCCTTCT) were used to check for genomic DNA contamination and as positive controls for the reverse transcription. Primers targeting the candidate gene for the putative cellulose synthase of *P. massiliensis* (forward: 5′ TCCACTCGACATGCAATCTT; reverse: 5′- AAAACACAAACCCGCTCTGC) and those targeting the cellulose synthase genes (KC466026.1 and XM_004335119.1) of *A. castellanii* (forward:5′GGGAGATCAACGACAACCTG; reverse: 5′-GTCCTCRGTCTGCGACTCGT) were designed using Primer3 (Koressaar and Remm, 2007). RNeasy MinElute Cleanup Kit (Qiagen) was used to purify total RNA according to the manufacturer’s recommendations. Total RNA was reverse-transcribed into cDNA by using the SuperScript VILO Synthesis Kit (Invitrogen, France). Then, qPCR was carried out on the cDNA with the LightCycler^®^ 480 SYBR Green 1 Master reaction mix (Roche Diagnostics, Mannheim, Germany), following the manufacturer’s temperature program with 62°C as primer hybridization and elongation temperature. Each experiment was performed in triplicate.

## Results

### *Pandoravirus massiliensis* Ultrastructure

**FIGURE 1:**
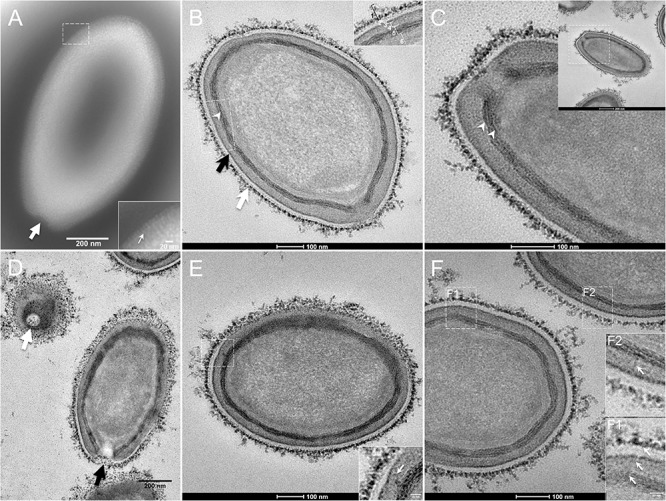
Transmission electron microscopy (TEM) of *Pandoravirus massiliensis*. **(A)** Negative staining of a *P. massiliensis* particle: the ostiole (arrow) is located at the apex of the particle. Peripheral thin fibers can be observed enwrapping the particle (thin arrow in boxed zoomed region). **(B)** Ultrathin section showing (i) the most-peripheral sugars depicted by ruthenium red aggregates (thick white arrow and level 1 in boxed zoomed region); (ii) a thin electron-dense membrane (thick black arrow and level 3 in boxed zoomed region) and more centrally (iii) a thick bundle of tubules (thin white arrowhead and level 5 in boxed zoomed region). **(C)** Two thick tubules compose the inner-most thick layer (arrowheads). **(D)** Two particles with ostioles cut transversally or perpendicularly (white and black arrows). **(E)** The inner-most thick tubules with thick protrusions (white arrow in boxed zoomed region) toward the outer thin electron-dense membrane. **(F)** Thin fibers (white arrows in F1 and F2 boxed zoomed regions) projecting from the inner-most thick tubules, crossing the outer electron-dense membrane and reaching the peripheral ruthenium-red stained sugars.

The study by transmission electron microscopy (TEM) of the ultrastructure of negatively stained purified *P. massiliensis* particles showed ovoid shaped virions with a mean maximal diameter of 1,230 ± 179 nm and a mean minimal diameter of 689 ± 114 nm (*n* = 10), these dimensions reaching up to 1,510 nm × 860 nm (Figure 1A). An ostiole with a concave shape could be observed at one apex of the particles (thick arrow in Figure 1A) and thin fibrillar structures were present around the particles (thin arrow in boxed zoomed region of Figure 1A).

Fixation with ruthenium red allowed good visualization of peripheral polysaccharides on ultrathin sections. We observed for all particles, from periphery to inside (Figure 1B): (i) peripheral sugars as depicted by ruthenium red aggregates (thick white arrow; level 1 in boxed zoomed region of Figure 1B), with electron-dense spikes originating from a thick layer of electron-dense aggregates; (ii) an electron-lucent space (level 2 in boxed zoomed region of Figure 1B); (iii) an electron-dense membrane (thick black arrow; level 3 in boxed zoomed region of Figure 1B); (iv) an homogeneous interspace (level 4 in boxed zoomed region of Figure 1B); (v) a thick electron-dense layer made of several tubules white arrowhead and level 5 in boxed zoomed region of Figure 1B; white arrowheads in Figure 1C); (vi) a smooth internal compartment, more dense at each apex (level 6 in boxed zoomed region of Figure 1B). Particles were cut along different planes, thus showing their different orientations, and the ostioles could be observed cut transversally or perpendicularly (Figure 1D). Structures originating from the thick inner tubular layer could be observed, such as thick tubules reaching the thin outer electron-dense membrane (arrow in boxed zoomed region of Figure 1E) or thin fibrils reaching the most peripheral ruthenium-red stained polysaccharides (arrows in boxed zoomed regions of Figure 1F).

**FIGURE 2:**
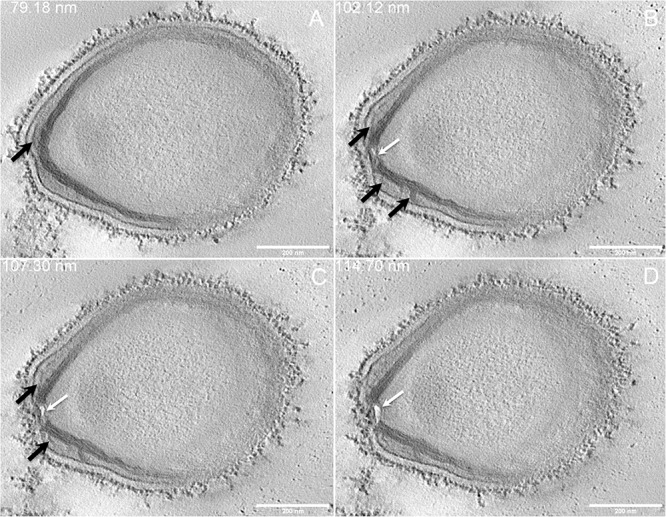
Electron tomography of *P. massiliensis* particle from **Movie 3**. **(A–D)** Single planes in the tomogram from **Movie 3** showing thick tubules protruding toward the periphery and the outer electron-dense membrane (black arrows); a thin tubule/membrane (white arrow) connects the thick tubules layers located on each side of the ostiole.

**FIGURE 3:**
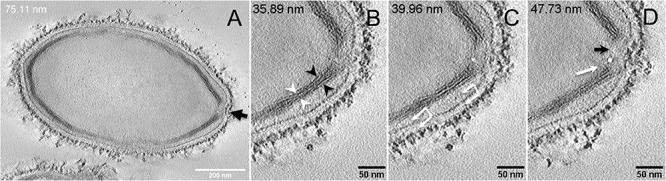
Electron tomography of *P. massiliensis* particle from **Movie 4**. **(A)** Single plane in the tomogram from **Movie 4** showing a whole *Pandoravirus* particle and its ostiole located at one apex (arrow). **(B)**. The inner-most layer is composed of two thick tubules well separated (black arrowheads) or contacting each other (white arrowheads). **(C,D)** Thin fibers (arrows) originating from the inner-most tubular thick layer projecting toward the peripheral sugars (white arrows, **C**), toward the inner core of the particle (white arrow, **D**) or at the level of the ostiole (black arrow, **D**).

**FIGURE 4:**
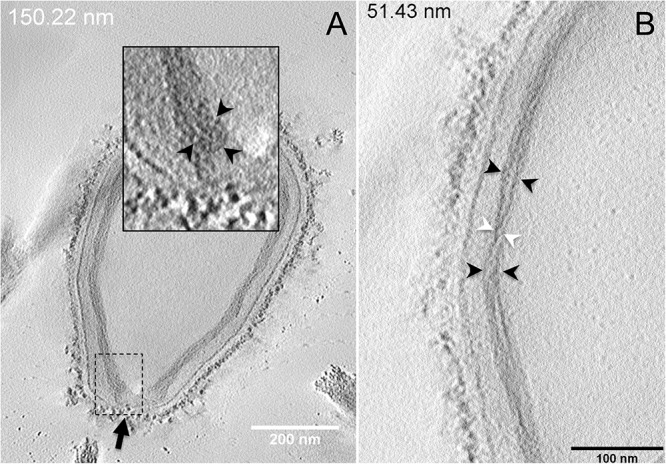
Electron tomography of *P. massiliensis* particles from **Movies 5** and **6**. **(A)** Single plane in the tomogram from **Movie 5** showing a *Pandoravirus* particle with its ostiole (black arrow). The magnified boxed region depicts a U-shaped thick tubule (black arrowheads) from the inner-most layer. **(B)** Single plane in **Movie 6** from the zoomed-in tomogram from **Movie 5** showing the helical structural arrangement of the two thick tubules (arrowheads) composing the inner-most layer of *Pandoravirus* particles with distant tubules (black arrowheads) or crossed tubules (white arrowheads).

Next, to get a more detailed ultrastructure, a three-dimensional (3D) electron tomography on 300 nm-thin sections of ruthenium-red fixed LR-White embedded *P. massiliensis* particles was performed. Eight tomograms were reconstructed. A tilt-series acquired at ×14,500 magnification (**Movie 1**) was used to reconstruct Tomogram 1, in which several particles can be observed (**Movie 2**). These particles were ovoid in shape with a homogeneous internal compartment, or non-ovoid with an electron-luscent internal compartment, suggesting deteriorated particles. Tomogram 2 (**Movie 3**) is a zoom-in on a particle from Tomogram 1. Selected Z-planes from tomogram 2 (Figure 2) illustrate structures originating from the inner thick tubular layer: thick structures with various diameters (black arrows in Figure 2) and thinner structures 2 nm thick at the level of the ostiole (white arrows in Figure 2). Tomogram 3 is shown in **Movie 4**. Selected Z-planes. From the tomograms and images, we observed that the inner structures have a tubular shape, with 8 nm in diameter (Figure 3A). From the tubular layer (Figure 3B), thin 2 nm-thick fibrils originate and cross the electron-dense outer membrane and project toward the peripheral sugars (Figure 3C), or toward the internal compartment on the opposite side of the ostiole (Figure 3D). Tomogram 4 (**Movie 5** and **Movie 6**) was chosen to illustrate the continuity between the two tubules from the inner-most tubular layer. These tubules can form a U-shape with no ending at the ostiole (Figure 4A). In addition, we found out that the two inner-most tubules can present a helicoidal arrangement. This finding is shown in tomogram 5 (**Movie 6**), which is a zoom-in sub-tomogram from tomogram 4. In Figure 4B, the two tubules from tomogram 5 are arranged as a helix, with locations where the two tubules are distant (black arrowheads in Figure 4B) and other locations where the two tubules are crossed (white arrowheads in Figure 4B). This helical structural arrangement of the inner-most thick tubular layer then became obvious when playing all tomograms acquired, and this organization could also be noticed when looking back at conventional ultra-thin sections without tomography. The two tubules were superposed forming a 10 nm-thick layer or alternatively distant of 30 nm. On average, this helical arrangement had a periodicity of 150 nm, from crossing points to the most distant location point of the tubules.

Subsequently, since the diameter and structure of inner-most tubules potentially forming helix resembled cellulose and chitin according to the literature, we hypothesized that the inner-most layer of the *P. massiliensis* particles consisted of cellulose and/or chitin.

### Cellulose Staining

**FIGURE 5:**
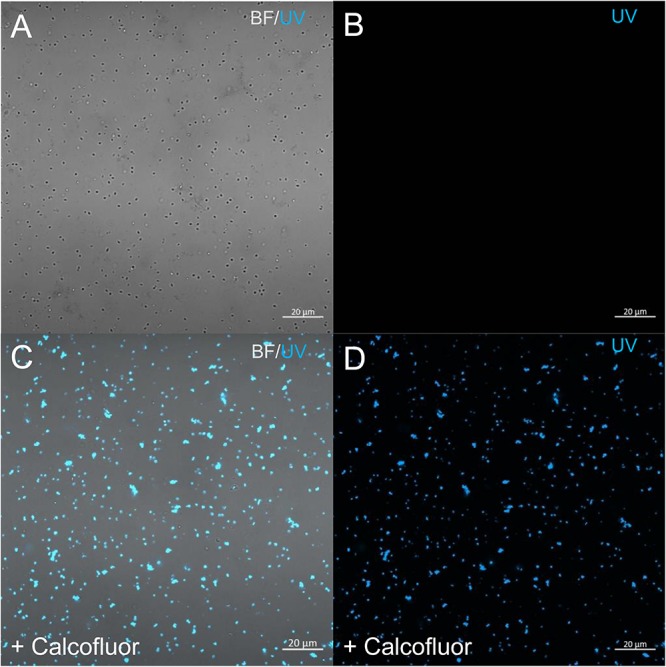
Confocal imaging of Calcofluor staining of *P. massiliensis*. **(A,B)** Control *Pandoravirus* particles. **(C,D)** Calcofluor-stained *Pandoravirus* particles. (BF: brightfield; UV: ultraviolet).

**FIGURE 6:**
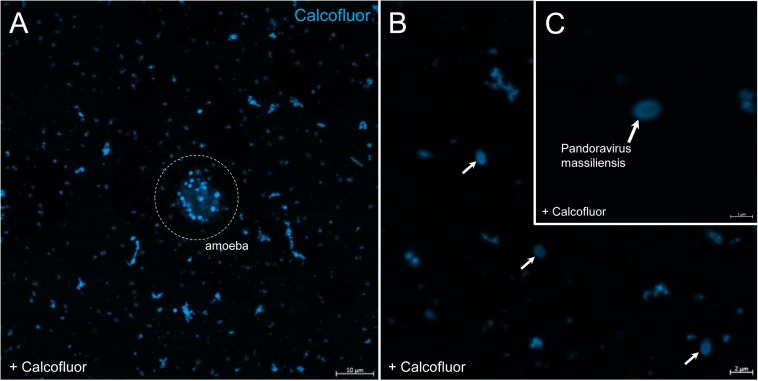
Confocal imaging of Calcofluor staining of *P. massiliensis*. **(A)** Pandoravirus-infected ameba and single *Pandoravirus* particles stained with Calcofluor white. **(B,C)** Calcofluor-stained *Pandoravirus* particles showing an intense peripheral calcofluor signal and a less-stained central region.

In order to check for cellulose content in *P. massiliensis* particles, a calcofluor white staining of viral particles smears and confocal imaging were performed. Negative control consisting in unstained particles did not show any fluorescence under UV illumination (Figures 5A,B). Inversely, calcofluor-stained particles were fluorescent under the same illumination (Figures 5C,D). Single in-liquid *P. massiliensis* particles were stained by calcofluor, as well as particles that remained in a few ameba still present after purification (Figure 6A). Zooming on individual particles showed that, on average, the fluorescence of the periphery of the particles seemed to be more intense than in the central region of the same particles (Figures 6B,C).

### Degradation of the *Pandoravirus* Particles by the Cellulase Treatments and Ultrastructure of Cellulase-Treated *Pandoravirus massiliensis* Particles

**FIGURE 7:**
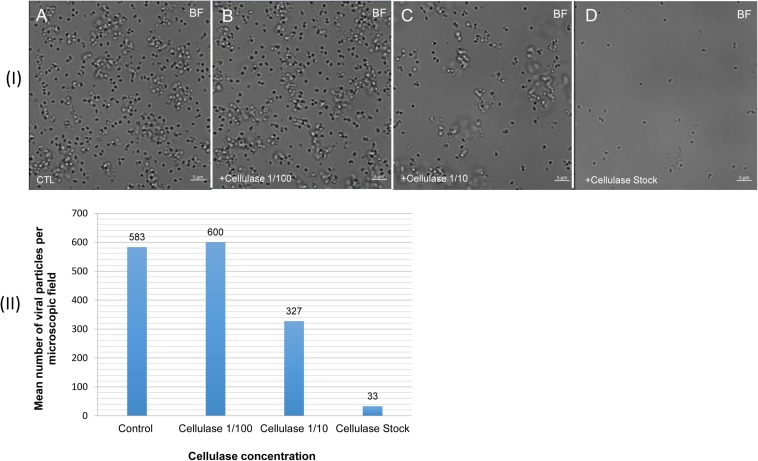
Confocal imaging of cellulase-treated *P. massiliensis* **(I)** and estimation of the mean number of particles of *P. massiliensis* per microscopic field of observation after cellulase treatment **(II)**. **(IA)** Control condition with untreated *P. massiliensis* particles. **(IB–ID)**: cellulase-treated *P. massiliensis* particles. **(II)** The mean number of particles of *P. massiliensis* per microscopic field of observation after cellulase treatment was assessed by the ImageJ software.

**FIGURE 8:**
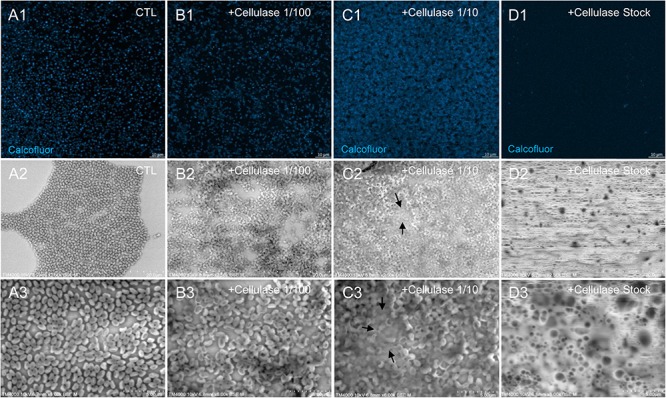
Confocal imaging of Calcofluor-stained cellulase-treated *P. massiliensis* virions and scanning microscopy of cellulose treated *P. massiliensis*. **(A1)** Control condition with untreated *P. massiliensis* particles stained with Calcofluor-white. **(B1,C1,D1)** Cellulase-treated *P. massiliensis* particles stained with Calcofluor-white. **(A2,A3)** Control condition with untreated *P. massiliensis*. **(B2,B3,C2,C3,D2,D3)** Cellulase-treated *P. massiliensis* particles imaged with scanning microscopy on two magnification.

The mean number of viral particles per microscopic field observed after cellulase treatment at high concentrations (stock solution and 1:10 dilution) by confoncal microscopy and assessed on 10 microscopic fields by the ImageJ software decreased in comparison with the negative control composed of the same viral sample not submitted to the enzymatic treatment (Figure 7). With a cellulase solution diluted at 1:10, the number of viral particles was slightly divided by two, and only ≈6% of the virions remained intact after a treatment by the cellulase stock solution (Figure 8). The scanning microscopy of *Pandoravirus* virions treated with the cellulase stock solution confirmed the progressive degradation of particles (Figures 8D2,D3). Moreover, calcofluor staining on viral particles treated by cellulase did not show any fluorescence under UV illumination (Figure 8D1). Besides, with a treatment with cellulase at 1:10 dilution, viral particles were partially digested, by forming a fluorescent matrix (black arrows in Figures 8C1–C3) suggesting a degradation of a cellulosic part of the viral particles. It should be noted that this result was less obvious in the most diluted solution of the enzyme (1:100) (Figure 8B1).

**FIGURE 9:**
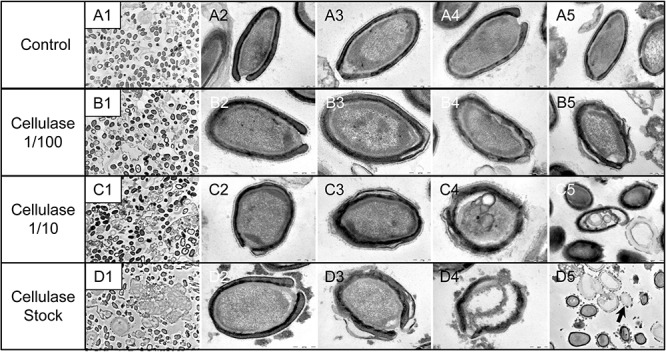
Transmission electron microscopy of cellulase-treated *P. massiliensis* particles. **(A1–A5)** Control condition with untreated *P. massiliensis* particles. **(B1–B5)** *P. massiliensis* particles treated with cellulase solution diluted at 1:100, **(B3–B5)** particles showed a defect of the envelope. **(C1–C5)** *P. massiliensis* particles treated with cellulase solution diluted at 1:10, **(C3–C5)** particles exhibited detachments of the different layers of the envelope. **(D1–D5)** *P. massiliensis* particles treated with cellulase solution stock. **(D3–D5)** Particles imaged in different stages of digestion from least to the most digested. **(D5)** “Ghost-like” particles presenting totally empty internal spaces and only a thin surrounding tegument (black arrow).

As compared to control, untreated *P. massiliensis* particles (Figures 9A1–A5), a degradation of the virions by the cellulase solution was observed by TEM (Figures 9B–D). Albeit this effect was not homogeneous between particles in all conditions, defective particles were observed with a dose-dependent effect of cellulase at the peripheral envelope and/or the internal compartment. Indeed, at 1/100 cellulase concentration, particles showed a defect of the envelope (Figures 9B3–B5) with the presence of electron-luscent regions between the thin electron-dense membrane and the thick bundle of tubules described in Figure 1. Detachments from the different layers were also observed in most of the affected particles (Figure 9B5). Accordingly, with 1/10 cellulase concentration treatment, particles exhibited electron-luscent regions at the level of the envelope as well as detachments of the different layers (Figures 9C3–C5), and also defects in the internal compartment with empty spaces and vacuoles (Figures 9C4,C5). This defect in the internal compartment was even more observable at stock concentration of cellulase (Figures 9D2–D4), with “ghost-like” particles presenting totally empty internal spaces and only a thin surrounding envelope (Figure 9D5). We also noticed the presence of debris and/or of an amorphous matrix in the ultra-thin sections of cellulase-treated *Pandoravirus* virions (Figures 9B1,C1,D1), in a dose-dependent manner coherent with the amorphous matrix seen by light microscopy and scanning electron microscopy under the same conditions (Figure 9), which may correspond to the debris of cellulase-digested particles.

The staining of the viral DNA fluorescent by the Hoechst dye performed after cellulase treatment showed fewer viral particles in the samples treated with cellulase stock solution and cellulase solution diluted at 1:10 than in the control or the particles treated with the cellulase solution diluted at 1:100 (Supplementary File S2). It should be noted that DNA of all viral particles that are in the correct plane of the confocal microscope field are fluorescent. Thus, hollow particles whose tegument was destroyed by the cellulase treatment did not content DNA.

Before the cellulase treatment, the TCID50 was 10^6.35^/mL, comparable to those of the control samples consisting in *Pandoravirus* particles incubated at 45°C during 48 h, for which the TCID50 was 10^6.51^/mL.

In the samples treated with the cellulase stock solution, the TCID50 was significantly lower, at 10^5.73^/mL.

### Cellulose Synthase Candidate Gene in the *Pandoravirus massiliensis* Gene Content

DELTA-BLAST analyses revealed a distant homology for the predicted gene 594 of *P. massiliensis* with the cellulose synthase domain bcsA of different bacteria such as *Escherichia coli*, *Shigella* sp., *Salmonella enterica* for the 30 best hits. These homologies were barely significant with the 10 best hits, identity percentages in amino acids varying between 24.7 and 23.9%, query coverages between 24 and 25%, and e-values ranging between 2e-15 and 3e-15 (Supplementary File S1). For this ORF594, no homology with a cellulose synthase was found by BLAST analyses neither against the nr database, nor against the CDD (Marchler-Bauer et al., 2017). Orthologs of this ORF594 were found by BLASTp analyses in all the 9 other pandoraviruses, with *e*-values varying from 3.41e-11 to 0.006; but none of them harbored the same bcsA domain when searched for by DELTA-BLAST analysis or in the CDD.

### Transcription of Candidate Gene of Cellulose Synthase of *Pandoravirus massiliensis* and Cellulose Synthase of *Acanthamoeba castellani*i

All qPCR experiments performed on *P. massiliensis* DNA using primers targeting the cellulose synthase candidate gene (ORF594) were positive, whereas all those performed on *A. castellanii Neff* DNA using the same primers were negative, which confirmed the specificity of the PCR system. Conversely, qPCR targeting the cellulose synthase gene of *A. castellanii* was positive on the amoebal DNA and negative on the viral DNA, confirming the amoebal specificity of these primers. All qPCR tests performed on the purified RNA extract after DNase treatment before the reverse transcription step were negative, indicating efficient DNA degradation.

**FIGURE 10:**
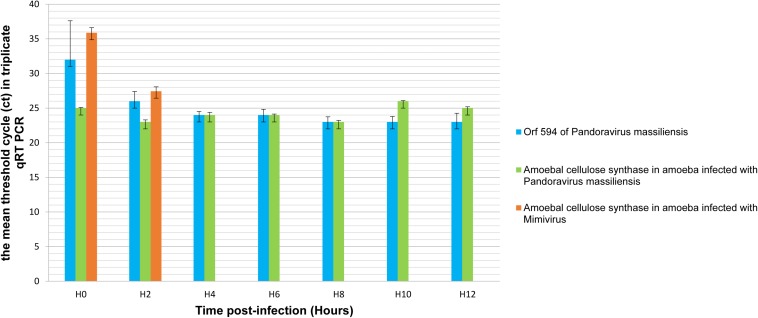
Representation of the mean threshold cycle (Ct) of triplicate qRT PCR on the RNA of *P. massiliensis* by targeting the predicted gene of the cellulose synthase (ORF594) and on RNA of *A. castellanii* infected once with *P. massiliensis* and a second time with Mimivirus by targeting the amoebal gene of the cellulose synthase, according to the time post-infection from 0 to 12 h post-infection.

qRT-PCR targeting *P. massiliensis* ORF594 were positive on the viral cDNA for samples collected during the whole viral cycle, indicating that this gene was transcribed. qRT-PCR targeting *A. castellanii* cellulose synthase gene were positive on the cDNA obtained from uninfected ameba and from ameba infected by *P. massiliensis* during the whole replicative cycle of *P. massiliensis* (Figure 10).

Conversely, qPCR experiments during the replicative cycle of Mimivirus in *A. castellanii* showed that the amoebal cellulose synthase was transcribed only during the very earlier time points of the cycle i.e., only at H0 and H2 post-infection (Figure 10).

These latter results show that the cellulose synthase gene of *A. castellanii* is transcribed both in uninfected ameba and in ameba infected with *P. massiliensis*.

## Discussion

We have determined here, by studying the ultrastructure of *P. massiliensis* virions by TEM after various treatments, that its viral integument, not previously characterized, was partially of a polysaccharide nature with a helical structure comparable to that of vegetable cellulose. Several markers of sugars (red ruthenium, calcofluor), the degradation of virions by cellulase and the use of appropriate negative controls clearly confirmed the cellulosic nature of the *Pandoravirus* viral tegument. The cellulose is the most common biologic macromolecule, biosynthesized by vegetals, algae, some bacteria, and by some marine animals as Ascidia (Nakashima et al., 2004; Helenius et al., 2006; Chen et al., 2016). It is also a major component of the cyst of *Acanthamoeba*, the unique host of pandoraviruses demonstrated to date (Magistrado-Coxen et al., 2019). It is composed of polymers of β (1,4) glucose subunits. Cellulosic chains are structured as microfibrils, which confer both resistance and plasticity to the vegetal walls, and also probably to the tegument of *P. massiliensis* virions. The bioinformatic analyses of the *P. massiliensis* genome possibly revealed a candidate gene (ORF594) for a cellulose synthase, which could be involved in the cellulose synthesis of the viral tegument. Indeed, a distant homology with the BcsA domain, which is one of the four catalytic subunits of the bacterial cellulose synthase was found (Wong et al., 1990). The domain bcsA polymerizes 5′-UDP-glucose to the cellulose polymer in formation (Zimmer, 2015). In the 9 other pandoraviruses, an ortholog for the ORF594 was found by BLASTp analysis. The experiments of qRT PCR revealed that this gene was transcribed during the viral cycle with an increased transcription from 2 h post-infection, and until 12 h post-infection, which could reinforce this first hypothesis.

However, the similarity with the bcsA domain is very low and this domain was not found in none of the 9 other pandoraviruses. Besides, the length of this domain bcsA found in bacteria ranges approximately around 800 amino-acids in length. The ORF594 is slightly smaller with a predicted encoded protein of only 135 amino-acids in length. The 3 other subunits of the cellulose synthase were not found in the *P. massiliensis* genome, neither by BLASTp nor by DELTA Blast analyses. Therefore, although this ORF594 is transcribed during the replicative cycle, the implication of the ORF594 in the synthesis of the cellulose is unlikely. One of the most amazing features of the pandoraviruses is the tremendous proportion of ORFans (genes without any homolog in the international sequence databases) and genes predicted to encode hypothetical proteins in their genome. These recently described viruses are so far to be exhaustively characterized. It has been shown that these hypothetical proteins are transcribed and translated (Philippe et al., 2013; Scheid, 2016; Aherfi et al., 2018; Legendre et al., 2018) suggesting that they have an efficient function for the virus. Among all these predicted genes, some of them could be implied in the synthesis of the cellulose, i.e., other unidentified cellulose synthase subunits, through synthesis by new enzymes or by a new metabolic pathway, unknown to date.

Given the lack of any bcsA domain in the orthologs of the ORF594 in other pandoraviruses and the lack of knowledge on the hypothetical proteins encoded by pandoraviruses, we could alternatively hypothesize that the virus could use for the synthesis of its particles tegument, the gene of the cellulose synthase of *A. castellanii* (Clarke et al., 2013). In a previous work, it has been shown that when *Acanthamoeba* was infected with a mimivirus, the transcription of the ameba felt dramatically and at 6 h after infection, the transcription became undetectable (Legendre et al., 2010). We observed herein, that the host gene of cellulose synthase was transcribed during the all the replication cycle of *P. massiliensis*, showing that the transcription of this amoebal gene is not impacted by the infection with *Pandoravirus*. Therefore, we can assume that the amoebal cellulose synthase could be diverted to the virus benefits, and could be involved in the synthesis of the cellulosic viral tegument. This gene was also found as transcribed, while *A. castellanii* were in the survival buffer, a buffer with the minimal components for the survival of ameba, and thus leading to the amoebal encystment. At this time, the ameba could start the synthesis of cellulose, a main component of the amoebal cyst.

In conclusion, we could determine herein the cellulosic nature of the tegument of *P. massiliensis*. Although a distant similarity was found with the catalytic subunit bcsA of the bacterial cellulose synthase, with a predicted ORF of *P. massiliensis*, this domain was not found in any other pandoravirus. These data suggest that the cellulose of the tegument of pandoraviruses could be probably the product of the host amoebal cellulose synthase. These results provide new important information about the previously unknown structure of the giant pandoraviruses, contributing to a best understanding of the biology of these complex viruses but also raising other fundamental questions about their interactions with their amoebal host.

## Data Availability Statement

The datasets generated for this study are available on request to the corresponding author.

## Author Contributions

DB and J-PB did the experiments and wrote the manuscript. FD and FG did the experiments. PC wrote the manuscript. SA supervised the experiments and wrote the manuscript. BL conceived the project, supervised the experiments, and wrote the manuscript.

## Conflict of Interest

The authors declare that the research was conducted in the absence of any commercial or financial relationships that could be construed as a potential conflict of interest.

## Supplementary Material

FILE S1 the alignment of the predicted gene 594 of *Pandoravirus massiliensis* with the cellulose synthase domain bcsA of different bacteria.

FILE S2 Confocal imaging of hoechst staining of *Pandoravirus massiliensis* particles after cellulase treatment. **(A)**: Control condition with untreated P. massiliensis particles stained with Hoechst. **(B,C,D)**: Cellulase-treated P. massiliensis particles stained with Hoechst.
