# Supplementary figures and images for: Evidence of a Cellulosic Layer in Pandoravirus massiliensis Tegument and the Mystery of the Genetic Support of Its Biosynthesis
Source: Front Microbiol. 2019 Dec 20;10:2932. doi: 10.3389/fmicb.2019.02932 (PMC6932959; doi:10.3389/fmicb.2019.02932)

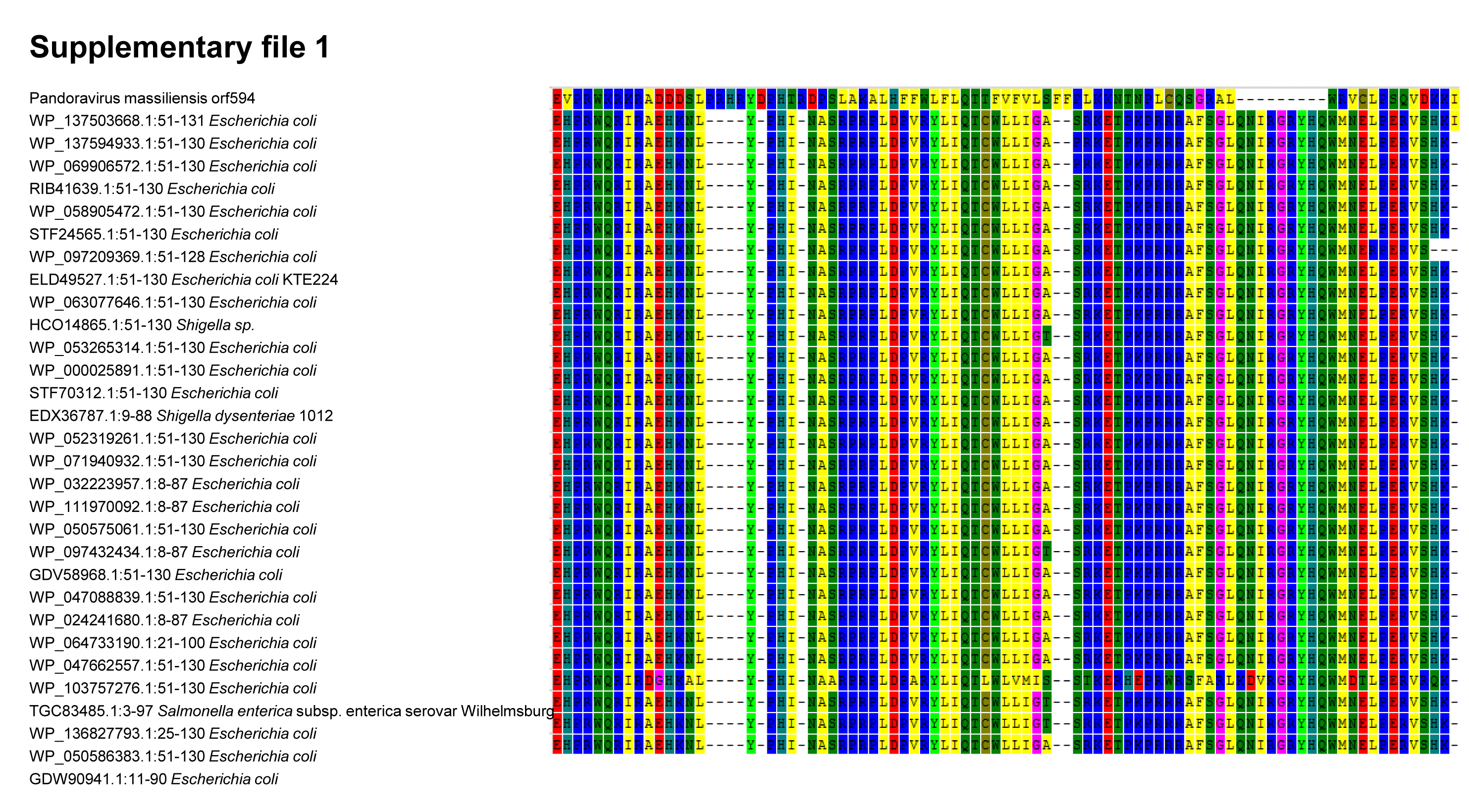

Supplement: FILE S1 — the alignment of the predicted gene 594 of Pandoravirus massiliensis with the cellulose synthase domain bcsA of different bacteria. [file Image_1.TIF]

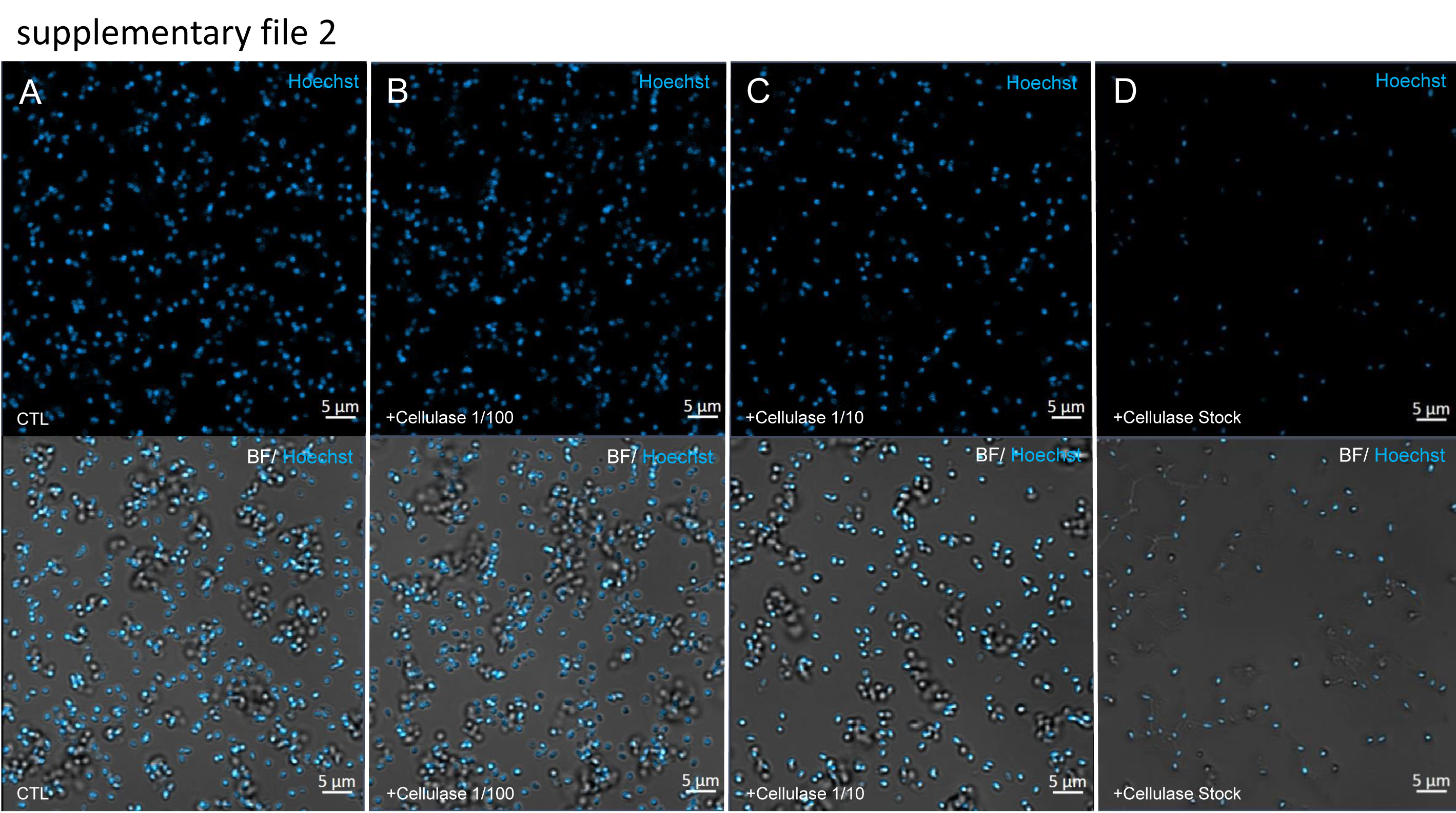

Supplement: FILE S2 — Confocal imaging of hoechst staining of Pandoravirus massiliensis particles after cellulase treatment. (A): Control condition with untreated P. massiliensis particles stained with Hoechst. (B,C,D): Cellulase-treated P. massiliensis particles stained with Hoechst. [file Image_2.TIF]
